# Unexpected Phosphaturic Mesenchymal Tumor of the Femoral Head

**DOI:** 10.3390/diagnostics13091632

**Published:** 2023-05-05

**Authors:** Hui Wang, Weijian Li, Wenxin Zhang, Peng Wang, Shen Wang, Ruiguo Zhang

**Affiliations:** Department of Nuclear Medicine, Tianjin Medical University General Hospital, No. 154 Anshan Road, Heping District, Tianjin 300052, China

**Keywords:** phosphaturic mesenchymal tumor, osteonecrosis of femoral head, mesenchymal tumor, CT, MRI

## Abstract

Osteonecrosis of femoral head (ONFH) is clinically common and easily diagnosed via imaging examination, especially when there is a definite cause, such as a fracture, long-term hormonotherapy, etc. However, some rare neoplastic lesions of the femoral head can mimic its image performance in some situations, leading to misdiagnosis. We present the case of a 57-year-old male with bone pain in the left hip joint that persisted for 2 years. CT and MRI images were performed and both were suggestive of ONFH. Unexpectedly, the histopathologic results of left proximal femur resection revealed the diagnosis of phosphaturic mesenchymal tumor (PMT), a rare mesenchymal tumor. His hip pain was obviously relieved after surgery, and the course of 1-year follow-up was uneventful.

We report the case of a 57-year-old man with bone pain, which mainly manifested in the left hip joint, persisting for two years. He came to our hospital because of his inability to walk, turn over, and perform other activities. The patient’s medical history involved having type 2 diabetes for 20 years. Physical examination showed significant tenderness to palpation of the left hip and positive Thomas sign, and the Gaenslen test could not be completed. Meanwhile, his laboratory data showed normal serum alkaline phosphatase (ALP) of 41 U/L (normal: 40–150), calcium 2.16 mmol/L (2.15–2.55), parathyroid hormone (PTH) 6.6 pmol/L (1.6–7.9) but mild hypophosphatemia of 0.72 mmol/L (0.81–1.45). Additionally, tumor markers were all negative. 

Computed tomography (CT) of the bilateral hips showed low-density bone destruction in the left femoral head (Figure 1A,B). The whole-body ^99m^Tc-MDP bone scintigraphy showed one focal intense area of tracer uptake in the left proximal femoral region (Figure 1C), corresponding to the lesion demonstrated on CT. No other abnormal distribution of ^99m^Tc-MDP was displayed. Subsequent magnetic resonance imaging (MRI) revealed the presence of an oval area with a hypointense signal on the T1-weighted image (Figure 2A,B) and a high–low mixed signal intensity on the T2-weighted image, which heterogeneously enhanced the contrast-enhanced MR scans (Figure 2C,D). The imaging characteristics of CT and MRI were suggestive of ONFH. Therefore, a resection of the left proximal femur and an artificial hip replacement was performed. Unexpectedly, histopathologic examination revealed spindle cells and vascularization, accompanied by some cartilage-like matrix that were positive for CD99 and vimentin and negative for cytokeratin and S100 protein—diagnostic of phosphaturic mesenchymal tumor (PMT). Results for Ki-67 highlighted a relatively high proliferation index (about 15%). His hip pain was obviously relieved after surgery, and the course of 1-year follow-up was uneventful.

Pathology results are the gold standard for validating a patient’s diagnosis of PMT. This patient’s features were as follows: (1) Clinical presentation: left hip bone ache for 2 years, unable to walk, mobility limitations, and so on. (2) Laboratory tests: ALP, blood calcium, PTH, liver and renal functions are all normal, blood phosphorus is slightly low, and so on. (3) Imaging manifestations: CT: hypodense destruction foci in the left femoral head; Whole-body bone imaging: left proximal femur restricted tracer concentration region; MR: T1-weighted picture oval low signal shadow, T2 image high and low mixed signal intensity, and so on. Generally, MRI has a high sensitivity for diagnosing ONFH, which is demonstrated by a limited linear low signal intensity under the cartilage on the T1 image and a bilinear sign-like pattern on the T2 image. Whole-body bone imaging usually shows a cold zone at the site of necrosis in the acute phase and a cold zone in the middle of a hot zone in the repair phase [1]. The patient’s clinical symptoms, laboratory tests, and some imaging characteristics may be similar to those of ONFH, although the MRI linear signal was not reflected, nor was the “donut sign” seen on the whole-body bone image. Be that as it may, ONFH was plausible and highly suspected.

Additionally, various causes of ONFH are associated with trauma, alcohol consumption, glucocorticoid application, diabetic patients, etc. When asked about their medical history, the patient denied any association with these conditions except for diabetes. Therefore, common conditions such as OFNH cannot be ruled out either.

These things considered, we suspected an incomplete subchondral bone fracture [1]. In addition to a similar clinical presentation, T1 and T2 images on MRI showed a low signal line in the subchondral bone and surrounding bone marrow oedema, with a lamellar high signal line in the fat-suppressed T2 image.

The diagnosis of PMT in this patient was an unexpected finding in the postoperative pathology. From the correct diagnostic standpoint, this patient presented with “ischemic necrosis of the femoral head” as a result of tumor destruction of the femoral head. Generally, due to the characteristic overexpression of fibroblast growth factor-23 (FGF-23), PMT possesses phosphaturic activity, which inhibits the conversion of 25 hydroxyvitamin D into osteotriol, as well as the absorption of phosphate by the renal tubules [2], and shows a series of laboratory abnormalities, such as increased urinary phosphate and decreased blood phosphate and phosphorus. Osteochondrosis PMT can affect any part of the body, in this case, the left femoral head. Unfortunately, the patient was not tested for FGF-23 at the time, but this does not preclude a normal FGF-23 value from denying PMT [3].

There is no better way to treat OFNH than surgical treatment, but secondary trauma from surgical intervention remains an unavoidable clinical problem [4]. Whether the lifestyle of patients can be improved after surgery is also a major concern. The most important concern for the patient was whether the highly sophisticated surgery would completely solve his mobility discomfort, the cost of surgery, and whether it was appropriate. Surgeons are also committed to addressing these issues. In contrast to ONFH, a more easily diagnosed benign femoral lesion, PMT is a rare mesenchymal tumor, which usually causes paraneoplastic syndromes, such as tumor-induced osteomalacia, due to hypophosphatemia, which is characterized by bone pain, muscle weakness, limited activity, and pathologic fractures and often occurs in bony (53%) or soft (45%) tissue sites along the axial or appendicular skeleton [5,6]. The medical burden and the cost of diagnosis of PMT for patients can be significant. Multiple different types of examinations and tests may be required to make an initial diagnosis. These include CT, MRI, blood tests, etc. As for the treatment, PMT usually requires surgery to remove and sometimes radiotherapy and/or chemotherapy [7]. Although the exact relationship between PMT and femoral head lesions is not yet clear, this phenomenon has attracted attention in the medical field. To avoid developing femoral head lesions, patients with PMT should undergo regular bone density checks and follow their doctor’s treatment recommendations. Other healthy habits to prevent femoral head lesions, such as avoiding excessive exertion and weight control, also apply to PMT patients. As for this patient, PMT or other tumors were not suspected prior to histological examination. This case serves as a reminder that the possibility of other diseases, such as PMT, should be considered when we encounter clinical manifestations and that laboratory tests are not quite consistent with the initial diagnosis of conditions such as ONFH in the femoral head, representing a challenging differential diagnosis.

## Figures and Tables

**Figure 1:**
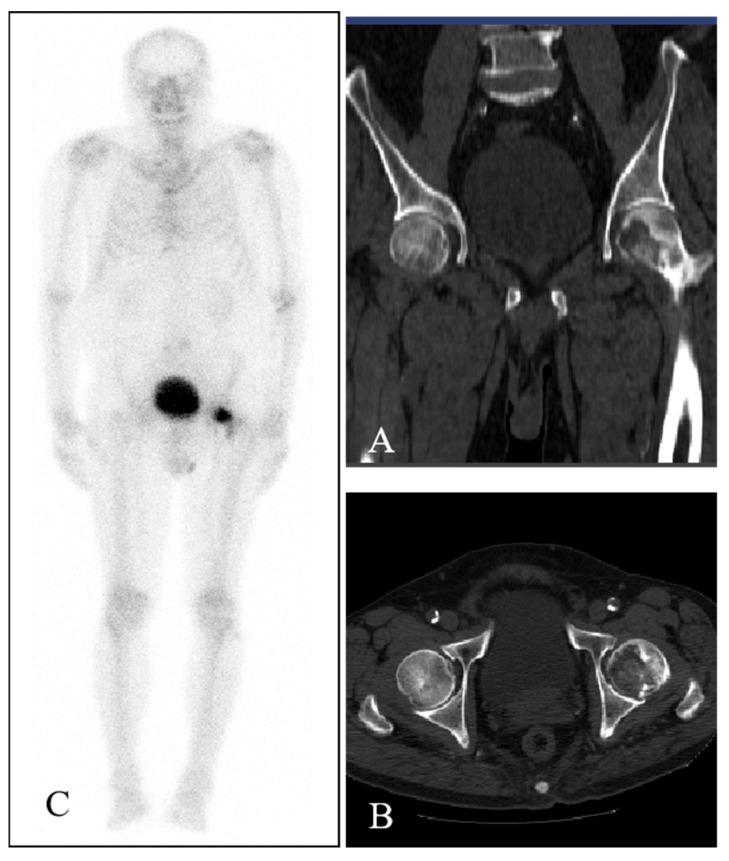
The coronal (**A**) and axial (**B**) CT images of bilateral hips showed a low density bone destruction in the left femoral head. The whole-body bone scintigraphy with ^99m^Tc-MDP showed one focal intense area of tracer uptake in the left proximal femoral region (**C**).

**Figure 2:**
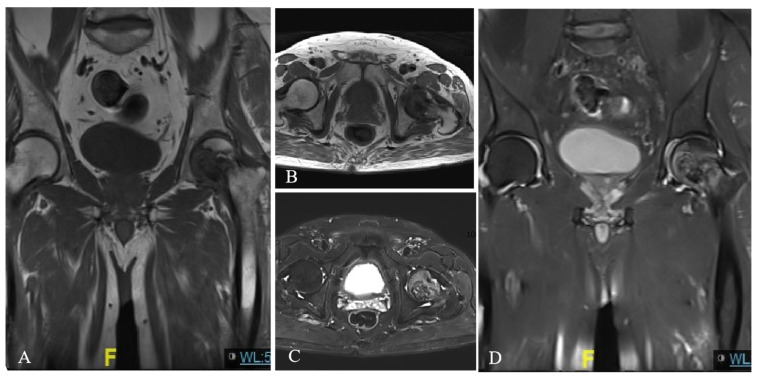
The coronal (**A**) and axial (**B**) T1-weighted images of bilateral hips showed a hypointense signal in the left femoral head, which showed the heterogeneous enhancement of the enhanced axial (**C**) and coronal (**D**) MR scans. “F” in Figure A and D represents "Front".

## Data Availability

In this article, all relevant data are presented. Further inquiries can be directed to the corresponding author.

## References

[1] Zhao D., Zhang F., Wang B., Liu B., Li L., Kim S.-Y., Goodman S.B., Hernigou P., Cui Q., Lineaweaver W.C. (2020). Guidelines for clinical diagnosis and treatment of osteonecrosis of the femoral head in adults (2019 version). J. Orthop. Transl..

[2] Dahir K., Zanchetta M.B., Stanciu I., Robinson C., Lee J.Y., Dhaliwal R., Charles J., Civitelli R., Roberts M.S., Krolczyk S. (2021). Diagnosis and Management of Tumor-induced Osteomalacia: Perspectives from Clinical Experience. J. Endocr. Soc..

[3] Benson J.C., Trejo-Lopez J.A., Nassiri A.M., Eschbacher K., Link M.J., Driscoll C.L., Tiegs R.D., Sfeir J., DeLone D.R. (2022). Phosphaturic Mesenchymal Tumor. AJNR Am. J. Neuroradiol..

[4] Cao H., Guan H., Lai Y., Qin L., Wang X. (2016). Review of various treatment options and potential therapies for osteonecrosis of the femoral head. J. Orthop. Transl..

[5] Wang X., Gao J., Han S., Li Y. (2019). Spinal phosphaturic mesenchymal tumors: Case report and literature review. J. Clin. Neurosci..

[6] Agaimy A., Michal M., Chiosea S., Petersson F., Hadravsky L., Kristiansen G., Horch R.E., Schmolders J., Hartmann A., Haller F. (2017). Phosphaturic Mesenchymal Tumors: Clinicopathologic, Immunohistochemical and Molecular Analysis of 22 Cases Expanding their Morphologic and Immunophenotypic Spectrum. Am. J. Surg. Pathol..

[7] Xiao X., Sun X., Ni P., Huang Y., Xie T. (2018). Phosphaturic mesenchymal tumor and related wound problem. Medicine (Baltimore).

